# A Symptom-Checker for Adult Patients Visiting an Interdisciplinary Emergency Care Center and the Safety of Patient Self-Triage: Real-Life Prospective Evaluation

**DOI:** 10.2196/58157

**Published:** 2024-06-27

**Authors:** Andreas Meer, Philipp Rahm, Markus Schwendinger, Michael Vock, Bettina Grunder, Jacopo Demurtas, Jonas Rutishauser

**Affiliations:** 1 In4medicine Inc Bern Switzerland; 2 Cantonal Hospital Baden Baden Switzerland; 3 Institute of Mathematical Statistics and Actuarial Science University of Bern Bern Switzerland; 4 Clinical Trial Unit Cantonal Hospital Baden and Medical Faculty University of Basel Baden Switzerland

**Keywords:** safety, telemedicine, teletriage, symptom-checker, self-triage, self-assessment, triage, triaging, symptom, symptoms, validation, validity, telehealth, mHealth, mobile health, app, apps, application, applications, diagnosis, diagnoses, diagnostic, diagnostics, checker, checkers, check, web, neural network, neural networks

## Abstract

**Background:**

Symptom-checkers have become important tools for self-triage, assisting patients to determine the urgency of medical care. To be safe and effective, these tools must be validated, particularly to avoid potentially hazardous undertriage without leading to inefficient overtriage. Only limited safety data from studies including small sample sizes have been available so far.

**Objective:**

The objective of our study was to prospectively investigate the safety of patients’ self-triage in a large patient sample. We used SMASS (Swiss Medical Assessment System; in4medicine, Inc) pathfinder, a symptom-checker based on a computerized transparent neural network.

**Methods:**

We recruited 2543 patients into this single-center, prospective clinical trial conducted at the cantonal hospital of Baden, Switzerland. Patients with an Emergency Severity Index of 1-2 were treated by the team of the emergency department, while those with an index of 3-5 were seen at the walk-in clinic by general physicians. We compared the triage recommendation obtained by the patients’ self-triage with the assessment of clinical urgency made by 3 successive interdisciplinary panels of physicians (panels A, B, and C). Using the Clopper-Pearson CI, we assumed that to confirm the symptom-checkers’ safety, the upper confidence bound for the probability of a potentially hazardous undertriage should lie below 1%. A potentially hazardous undertriage was defined as a triage in which either all (consensus criterion) or the majority (majority criterion) of the experts of the last panel (panel C) rated the triage of the symptom-checker to be “rather likely” or “likely” life-threatening or harmful.

**Results:**

Of the 2543 patients, 1227 (48.25%) were female and 1316 (51.75%) male. None of the patients reached the prespecified consensus criterion for a potentially hazardous undertriage. This resulted in an upper 95% confidence bound of 0.1184%. Further, 4 cases met the majority criterion. This resulted in an upper 95% confidence bound for the probability of a potentially hazardous undertriage of 0.3616%. The 2-sided 95% Clopper-Pearson CI for the probability of overtriage (n=450 cases,17.69%) was 16.23% to 19.24%, which is considerably lower than the figures reported in the literature.

**Conclusions:**

The symptom-checker proved to be a safe triage tool, avoiding potentially hazardous undertriage in a real-life clinical setting of emergency consultations at a walk-in clinic or emergency department without causing undesirable overtriage. Our data suggest the symptom-checker may be safely used in clinical routine.

**Trial Registration:**

ClinicalTrials.gov NCT04055298; https://clinicaltrials.gov/study/NCT04055298

## Introduction

In potentially critical situations, clinical warning signs and symptoms may be considered too late by patients due to a lack of professional triage [1]. In this context, various initiatives have been launched to improve outpatient emergency care and the population’s access to a low-threshold initial medical assessment [2]. Symptom-checkers, which enable medical self-triage, have recently been introduced for this purpose. Such tools could assist the increasing number of persons without ready access to a primary care physician, for example, migrants or young persons who had been previously healthy. If implemented in settings outside the hospital, that is, at home or work, tools for efficient and safe self-triage could help avoid unnecessary emergency hospital visits, thus contributing to reducing overcrowding and costs.

To fulfill the regulatory requirements [3] and to be used as a part of standard care, the appropriateness and safety of these instruments must be evaluated in concrete clinical settings with real patients [4-6].

Appropriate care results from adequate triage and treatment, while inappropriate care may lead to unsuitable or even dangerous health care delivery. The concept of appropriateness hence includes a widespread range of quality aspects, of which safety is only one. The difficulty of assessing appropriateness in health care and of gaining agreement between clinicians on acceptable and safe care is highlighted by different authors [7,8]. When assessing the appropriateness of medical triage, the question “Was the decision right” suggests that there is merely one single correct triage decision. This question does not appropriately reflect the complex interaction of clinical, social, and environmental factors in medical decision-making. Rather, physicians should consider a range of appropriate triage decisions to guide their actions. Safety is an essential quality attribute of a medical service. In contrast to the idea of appropriateness, the concept of safety focuses on the risk of a specific conduct. When asking about the safety of a symptom-checker, a risk-based approach should be taken, and safety should encompass possible risks to a patient’s health and life [3].

An evaluation of 23 symptom-checkers using 45 patient vignettes concluded that most symptom-checkers were deficient in both appropriate triage and correct diagnosis [9]. However, the study did not comment on the safety of the tested devices. A review paper including 14 studies found inconsistent evidence regarding the triage and diagnostic appropriateness of symptom-checkers for common health problems. The average appropriateness of triage ranged from 27% to 92%. This paper did not specifically evaluate the safety of symptom-checkers [5]. Another review paper cited only 6 studies that analyzed the safety of symptom-checkers [10]. These studies were mostly short-term and included samples that were too small and heterogeneous to make reliable statements about safety.

Given the present shortage of data on self-triage, we aimed to investigate the safety of a newly developed symptom-checker (SMASS; Swiss Medical Assessment System) in a concrete clinical setting with patients seeking emergency care.

## Methods

### Study Design

Before the inclusion of the first patient, this study was registered (ClinicalTrials.gov identifier: NCT04055298).

This study was performed between November 25, 2019, and May 1, 2020, at the walk-in clinic and interdisciplinary emergency department (WIC/ED) of the cantonal hospital of Baden, Switzerland. The WIC/ED is open 24 hours a day, 365 days a year and treats about 55,000 patients annually. Patients are routinely triaged by a nurse using the Emergency Severity Index (ESI) [11]. ESI 1-2 patients are treated in the ED, while ESI 3-5 patients are treated in the WIC.

The symptom-checker used in this study (SMASS in the pathfinder version, release 4.1.12) was developed by in4medicine, Inc. The first author (author AM) is the chief executive officer and founder of this company. To minimize bias, a majority of independent researchers were involved in this study, including establishing the protocol and all practical aspects of the trial. No employee of in4medicine took part in the actual conduct of the trial. Data analysis and statistical calculations were performed by an independent biostatistician. This study was independently monitored by the clinical trial unit of the Medical Faculty of the University of Bern.

The SMASS pathfinder symptom-checker is a medical device class I under the Medical Device Directive and medical device class IIb under the Medical Device Regulation. The Conformité Européenne declaration of conformity to the Swiss Agency for Therapeutic Products (Swissmedic) was made on June 4, 2018. The symptom-checker is a web-based software that aims to support health professionals and laypersons in the structured documentation and assessment of health problems and to advise users about possible medical assessment steps and treatment measures. It is based on a computerized neural network that incorporates extensive data from scientific studies, guidelines, and expertise from various professional boards of specialists in the field of prehospital medical triage. The symptom-checker provides digitalized questionnaires of 125 frequent reasons for consultations (eg, fever, cough, and abdominal pain) and their associated red flags. Based on the triage result, a report including patient gender, age group, symptoms, medical history, and recommendations as to the appropriate time-to-treat and point-of-care is provided. Depending on the presence of red flags, the symptom-checker assigns the clinical condition of the patient to a triage level (Tables 1 and 2). If 5 or more assessment questions are answered as “unclear,” the user is notified that the software cannot provide targeted triage advice and that the patient should seek immediate consultation with a physician concerning his or her medical complaints.

**Table 1:** Triage levels as recommended to the patient by the symptom-checker. Recommendations are given regarding time-to-treat (emergency, immediately, today, later, and unclear) and point-of-care (ambulance, hospital, physician, call center, pharmacy, self-care, and unclear).

	Ambulance	Hospital	Doctor	Call center	Pharmacy	Self-care	Unclear
Emergency	16	15	—^a^	—	—	—	—
Immediately	14	13	12	—	—	—	—
Today	—	11	10	8	6	4	—
Later	—	—	9	7	5	3	1
Unclear	—	—	—	—	—	2	0

^a^Not applicable.

**Table 2:** Recommended actions to be taken by the patient, as defined by triage levels (left column). Levels range from 0 (lowest level) to 16 (highest level). Interpretations of the triage level and measures to be taken are specified in the right column.

Triage level	Name	Recommended action
Level 16	Emergency ambulance	CPR^a^/CPR readiness. There is a potentially life-threatening condition. Medical treatment must be given now. Alert the emergency services via the number 144.
Level 15	Emergency hospital	CPR/CPR readiness. There is a potentially life-threatening condition. Medical treatment must be given now. Alert the emergency services via the number 144. Medical treatment should be provided at a hospital.
Level 14	Immediately ambulance	Medical treatment does not allow any delay. Treatment should be given immediately. Alert the emergency services via the number 144.
Level 13	Immediately hospital	Medical treatment does not allow any delay. Treatment should be given immediately. Medical treatment should be provided at a hospital.
Level 12	Immediately doctor	Medical treatment does not allow any delay. Treatment should be given immediately. Medical treatment should be provided by a registered doctor ^b^.
Level 11	Today hospital	Medical treatment does not have to take place immediately, but should not be delayed until tomorrow or over the weekend. Medical treatment should take place within the next 24 hours. Medical treatment should be provided at a hospital.
Level 10	Today doctor	Medical treatment does not have to take place immediately, but should not be delayed until tomorrow or over the weekend. Medical treatment should take place within the next 24 hours. Medical treatment should be provided by a registered doctor ^b^.
Level 9	Later doctor	Medical treatment is not urgent. If the symptoms do not subside in the next 2 days, treatment by a doctor is indicated. Medical treatment should be provided by a registered doctor ^b^.
Level 8	Today call center	Medical treatment does not have to take place immediately, but should not be delayed until tomorrow or over the weekend. Medical treatment should take place within the next 24 hours. The affected person should be advised by a telemedicine center on how to proceed.
Level 7	Later call center	Medical treatment is not urgent. If the symptoms do not subside in the next 2 days, treatment by a doctor is indicated. The affected person should be advised by a telemedicine center on how to proceed.
Level 6	Today pharmacy	Medical treatment does not have to take place immediately, but should not be delayed until tomorrow or over the weekend. Medical treatment should take place within the next 24 hours. The affected person should be advised at a pharmacy on how to proceed.
Level 5	Later pharmacy	Medical treatment is not urgent. If the symptoms do not subside in the next 2 days, treatment by a doctor is indicated. The affected person should be advised at a pharmacy on how to proceed.
Level 4	Today self-care	Medical treatment does not have to take place immediately, but should not be delayed until tomorrow or over the weekend. Medical treatment should take place within the next 24 hours. The complaints can be treated independently by simple measures.
Level 3	Later self-care	Medical treatment is not urgent. If the symptoms do not subside in the next 2 days, treatment by a doctor is indicated. The complaints can be treated independently by simple measures.
Level 0-2	Unclear	The survey contains too many ambiguities. A targeted initial assessment is not possible.

^a^CPR: cardiopulmonary resuscitation.

^b^For example, family doctor, family doctor substitute, family doctor emergency service, or suitable specialist.

All patients aged ≥18 years attending the WIC/ED between 8 AM and 5 PM were eligible. Exclusion criteria included aged <18 years; ESI 1 patients requiring immediate, life-saving intervention; inability to use a tablet PC; inability to communicate in German, French, Italian, or English; inability or unwillingness to give written informed consent and follow the procedures of this study; known or suspected noncompliance; known drug or alcohol abuse; the presence of symptoms or complaints not encompassed by the symptom-checker database (eg, long-lasting hiccups, hair loss).

After instruction by the study’s staff and providing written informed consent, the participants independently assessed their health status and complaints as instructed by the symptom-checker on a tablet PC. They were subsequently evaluated and treated by routine medical staff.

In primary care, medical triage decisions usually have to be based solely on the patient’s symptoms. We have chosen experts independent of the treatment (panels A, B, and C) as evaluators to ensure that the triage decision is based purely on the symptoms of this study’s patients. Including treating physicians as comparators in this study could have influenced the triage decision by additional information (physical examination and diagnostic test results).

Our evaluation of the symptom-checker focused on safety, as this is an essential quality attribute of a medical device [3]. To reflect the highly individual nature of medical decision-making, which usually results in low interrater reliability [12-14], an independent team of experienced physicians engaged in a stepwise evaluation procedure in which each case that was classified as undertriaged by panel A experts was assessed by several experts.

A research assistant and 3 external interdisciplinary panels of board-certified physicians were involved in the evaluation process (panel A, 5 experts; panel B, 2 experts; panel C, 5 experts). Except for one of the 12 panelists (author BG), they were not affiliated with in4medicine, Inc. None of them took part in the conduct of this study. For every patient, the symptom-checker issued a report summarizing the clinical information. Patients and panelists were unaware of the triage-level recommendations (time-to-treat and point-of-care) made by the symptom-checker. All reports were first assessed by members of panel A, who adjudicated an appropriate range of triage levels to every case. The research assistant then compared the adjudication of the panel A experts with the recommendation issued by the symptom-checker. If the comparison showed that the recommendation of the symptom-checker was below the appropriate range of triage levels determined by the rater of panel A, hence was undertriaged, the case was assigned to panel B. In 80 instances, panelists erroneously examined the same cases twice and concluded on diverse triage recommendations. In these cases, the first of the 2 recommendations was used for the analysis.

The evaluation procedure was repeated by panel B. Each of the 2 panelists evaluated all diverging cases. If the case was undertriaged according to 3 experts (1 expert from panel A and 2 experts from panel B), the case was subsequently analyzed by panel C.

Each member of panel C individually assessed the clinical safety of the triage decision based on the complete structured reports generated by the symptom-checker as well as the WIC/ED’s redacted discharge reports. Each of the 5 panelists decided individually on potentially hazardous undertriage. In a modified Delphi process, the panelists first individually adjudicated potentially hazardous undertriage on a 4-point Likert scale. Possible ratings were “unlikely,” “rather unlikely,” “rather likely,” and “likely” that the patient was exposed to a risk to life or health. If the panelists subsequently reached a consensus that the triage of the symptom-checker was “rather likely” or “likely” exposing a patient to a risk to life or health, the case was considered a potentially hazardous undertriage (consensus criterion). As a complement to the original analysis plan, a modified criterion for potentially hazardous undertriage was evaluated, defined as a majority of panel C members judging a risk to life or health as “rather likely” or “likely” (majority criterion).

The primary analysis consisted of the calculation of the 95% upper Clopper-Pearson confidence bound for the probability of undertriage resulting in a risk to life or health (potentially hazardous undertriage). To confirm the safety of the symptom-checker, this upper confidence bound should lie below 1%. For the sample size calculation, we assumed that a 20% probability of failure to meet this criterion is acceptable for a true probability of potentially hazardous undertriage of no more than 0.5%. This is equivalent to requiring a 1-sided test at level 5% to show that the probability of potentially hazardous undertriage is below 1% with a power of 80%, assuming that the true probability is 0.5%. This resulted in a minimal sample size of 2185 patients. Accounting for an estimated rate of 2% “unclear” responses, at least 2230 patients were planned to be included. Secondary analyses included central 95% Clopper-Pearson CIs for the further probabilities, based on corresponding empirical proportions. The software R (version 4.2.0; R Foundation for Statistical Computing) was used for the statistical evaluations.

### Ethical Considerations

This study was approved by the competent ethics committee (Ethikkommission Nordwest- und Zentralschweiz EKNZ, project ID 01784) and was conducted per the most recent version of the Declaration of Helsinki, complying with International Council for Harmonisation of Technical Requirements for Pharmaceuticals for Human Use—good clinical practice and International Organization for Standardization European Norm 14155 (clinical investigation of medical devices for human subjects—good clinical practice) as well as with applying national legal and regulatory requirements. All patients gave written informed consent to participate in this study. They did not receive any financial or other compensation. Patients were anonymized upon data collection. Discharge notes studied by panel C were redacted.

Generative artificial intelligence was not used in any portion of this paper’s writing.

## Results

**Figure 1:**
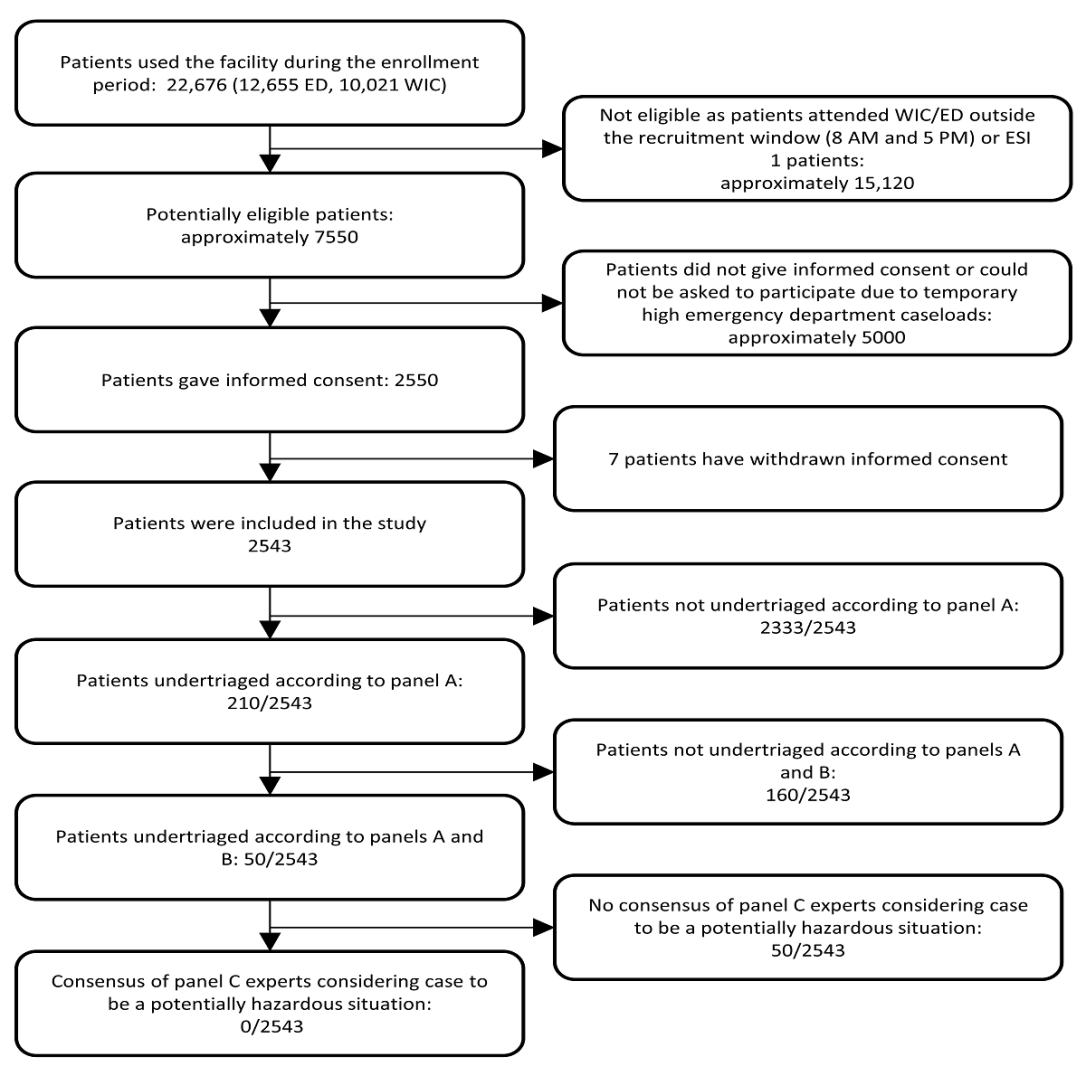
Flow of patients through this study and triage assessment steps by expert panels A, B, and C. ED: emergency department; ESI: Emergency Severity Index; WIC: walk-in clinic.

The baseline characteristics of the participants are shown in Table 3 and the recommendations obtained by the symptom-checker in Table 4. Figure 1 shows the flow of analyses by panels A-C.

**Table 3:** Characteristics of the study population (N=2543).

Characteristics			Participants, n (%)
**Age (years)**			
	18-49	1397 (54.94)	
	50-65	668 (26.27)	
	66-80	360 (14.16)	
	>80	118 (4.64)	
**Gender**			
	Female	1227 (48.25)	
	Male	1316 (51.75)	
**Reason for encounter (15 most frequent)**			
	Stomach pain	287 (11.26)	
	Chest pain	168 (6.61)	
	Lumbar back pain	144 (5.66)	
	Urinary tract problems	124 (4.88)	
	Trauma or fall	121 (4.76)	
	Headache	90 (3.54)	
	Dizziness	87 (3.42)	
	Wound or skin injury	82 (3.22)	
	Foot injury (caused by an accident)	81 (3.19)	
	Leg problems	74 (2.91)	
	Breathlessness	69 (2.71)	
	Cold or influenza infection	64 (2.52)	
	Finger injury (caused by an accident)	55 (2.16)	
	Knee injury (caused by an accident)	51 (2.01)	
	Hand injury (caused by an accident)	43 (1.69)	

**Table 4:** Distribution of cases according to the various triage levels, as defined in (N=2543).

Triage level	Participants, n (%)
Emergency ambulance	57 (0.02)
Emergency hospital	142 (0.06)
Immediately ambulance	2 (0)
Immediately hospital	685 (0.27)
Immediately doctor	844 (0.33)
Today hospital	3 (0)
Today doctor	579 (0.23)
Later doctor	36 (0.01)
Today call center	26 (0.01)
Later call center	60 (0.02)
Today pharmacy	0 (0)
Later pharmacy	30 (0.01)
Today self-care	0 (0)
Later self-care	77 (0.03)
Unclear	2 (0)

In 210 (8.26%) of the 2543 cases, the recommendation issued by the symptom-checker was below the range of appropriate triage levels defined by the panel A experts and therefore undertriaged. Further, 50 (1.96%) of these 210 patients were equally undertriaged according to panel B. However, for none of these 50 patients did panel C reach a consensus that the undertriage was potentially hazardous. This resulted in an upper 95% confidence bound for the probability of a potentially hazardous undertriage of 0.1184%. If the criterion for potentially hazardous undertriage was defined as a majority of panel C members considering life-threatening or harmful self-triage “rather likely” or “likely,” 4 of the 50 cases fulfilled this criterion. This resulted in an upper 95% confidence bound for the probability of a potentially hazardous undertriage of 0.3616%.

Table 5 shows the adjudication of potentially hazardous undertriage for all 50 cases evaluated by the experts of panel C.

**Table 5:** Distribution of assessment for potentially hazardous undertriage for all 50 cases, as adjudicated by each of the 5 members of panel C.

Case number	Unlikely	Rather unlikely	Rather likely	Likely
Case 1	2	3	0	0
Case 2	5	0	0	0
Case 3	3	2	0	0
Case 4	3	2	0	0
Case 5	4	0	1	0
Case 6	5	0	0	0
Case 7	2	2	1	0
Case 8	4	1	0	0
Case 9	0	2	2	1
Case 10	4	0	1	0
Case 11	5	0	0	0
Case 12	5	0	0	0
Case 13	1	1	2	1
Case 14	4	1	0	0
Case 15	2	2	1	0
Case 16	5	0	0	0
Case 17	4	1	0	0
Case 18	5	0	0	0
Case 19	5	0	0	0
Case 20	5	0	0	0
Case 21	3	1	1	0
Case 22	2	1	1	1
Case 23	3	1	1	0
Case 24	5	0	0	0
Case 25	5	0	0	0
Case 26	5	0	0	0
Case 27	5	0	0	0
Case 28	5	0	0	0
Case 29	2	1	2	0
Case 30	4	0	1	0
Case 31	5	0	0	0
Case 32	2	1	1	1
Case 33	4	1	0	0
Case 34	4	1	0	0
Case 35	5	0	0	0
Case 36	0	2	3	0
Case 37	4	1	0	0
Case 38	5	0	0	0
Case 39	5	0	0	0
Case 40	2	1	1	1
Case 41	1	3	1	0
Case 42	5	0	0	0
Case 43	5	0	0	0
Case 44	5	0	0	0
Case 45	5	0	0	0
Case 46	5	0	0	0
Case 47	5	0	0	0
Case 48	1	0	4	0
Case 49	4	1	0	0
Case 50	5	0	0	0

The central (2-sided) 95% Clopper-Pearson CI for the probability of undertriage according to panel A is 7.22% to 9.40%. The central (2-sided) 95% Clopper-Pearson CI for the probability of overtriage according to panel A (450 cases, 17.69%) is 16.23% to 19.24%.

For the 50 out of 2543 cases that were undertriaged according to the judgments of panels A and B, the central (2-sided) 95% Clopper-Pearson CI for the corresponding probability is 1.539% to 2.688%.

The central (2-sided) 95% Clopper-Pearson CI for the probability of a potentially hazardous undertriage for the consensus criterion (0 out of 2543 cases) is 0% to 0.1458% and 0.0431% to 0.4045%, according to the majority criterion (4 out of 2543 cases).

## Discussion

### Principal Findings

Our study corroborates the safety of the SMASS pathfinder symptom-checker for medical self-assessment of acute complaints in a real-life clinical setting. A stepwise evaluation of 2543 consecutive patients by 3 independent expert panels yielded no cases of potentially hazardous undertriage when the consensus criterion was applied and 4 cases when the majority criterion was applied.

In a systematic literature search, we found insufficient evidence from comparatively small studies for the safe use of symptom-checkers in clinical routine (Demurtas et al, unpublished data, 2021). Further, 1 study with 825 patients showing “exactly matched” triage in 52.6% has been published in abstract form only [15]. Another study yielded correct triage in only 50%-74% of cases [16]. A third study, from Germany, evaluated the safety of urgency advice provided to 378 patients at an interdisciplinary ED by a symptom-checker [17], showing undertriage in 34 (8.9%) and overtriage in 216 (57.1%) cases. A potentially hazardous situation was identified in 20 (5.3%) cases. This figure appears considerably higher than our finding, although an interrater variability was not taken into account in the German study. Another study aimed to analyze the performance of a clinical decision support system that allowed patients to self-triage in the ED of a university hospital. The authors concluded that the self-triage device was safe, as the assessments by the system and the physicians were congruent concerning the classification as an emergency. However, in contrast to our study, the risk to life or health was not assessed [18].

In the absence of a broad study base, we cannot compare our results with previous, similarly designed studies for symptom-checkers. In contrast, medical telephone triage has been extensively evaluated during the last 25 years [19-24] and has gained broad clinical support, despite ambivalent conclusions regarding safety.

In a systematic review analyzing 13 observational studies and 10 studies that simulated high-risk patients, safe triage was found to be 46% to 97% [25]. Another systematic review involving computer-assisted telephone triage in urgent care [26] pointed out 4 studies that indicated potential undertriage errors [27-30]. Notably, hospitalization rates of patients who were advised to seek nonurgent care ranged from 9.2% to 48%. Potentially life-threatening situations emerged in 0.84% of cases according to 1 study [29].

We have previously investigated the safety of computer-assisted telephone triage in 208 patients with non–life threatening conditions consulting the ED at a university hospital [31]. We found poor agreement between the assessments by the call center, the emergency physician, and the general practitioners who later cared for the patients. In 1 case, a risk to health or life was found.

The Cochrane Collaboration in their 2004 systematic review on telephone triage concluded that insufficient data existed regarding safety [32]. In light of the available information, the results of our study compare favorably to the published data on telephone triage.

Our study has several strengths and weaknesses. We included a large number of patients in a real-world clinical setting. In addition, this study’s design enabled us to eliminate the low interrater reliability of medical triage decisions by having 3 independent expert panels. This allows robust conclusions about the safety of the evaluated symptom-checker.

For reasons of feasibility, we performed our study in a hospital setting, where patients were triaged to the WIC or ED according to ESI criteria. Thus, a wide variety of cases could be assessed. On the other hand, the symptom-checker was not used in a setting outside the hospital, limiting generalizability. However, presenting symptoms largely overlap with those encountered in primary care, and a potential selection bias toward more severe cases would support the conclusion on the device’s safety if it were used in primary care.

A potential limitation of our study is its single-center design. However, the Cantonal Hospital Baden serves a mixed urban and rural population of approximately 300,000 people and offers all medical services except cardiac surgery and neurosurgery. We therefore believe that the patient sample in our study is fairly representative of the general population.

The total number of patients frequenting the WIC and ED during the time of recruitment was 22,676; thus, only approximately 11% of them participated in this study, potentially resulting in selection bias. Due to limited resources, inclusions were possible only during the daytime, leaving approximately 7550 potential participants. Further, 1.5% (340/22,676) were ESI 1 patients, who were not eligible for this study. It could be speculated that patients visiting an ED at night time might be more seriously ill than those during the daytime. This potential bias would make our cohort more comparable to a setting in primary care.

In our study, we have focused on the safety of the symptom-checker. A possible limitation may have resulted from the fact that each case was initially assessed by a single member of panel A. This could have precluded passing a potentially hazardous case to panel B. While maximum patient safety may theoretically be desirable, it should be weighed against the disadvantages of overtriage, notably inefficiency, unnecessary referrals, and a higher risk of overmedicalization, all of which increase costs. In our study, the overtriage rate after assessment by panel A was 17.69% (450 cases). This figure is comparable to published rates of overtriage by teleconsultation and teletriage, which range from 12% to 57% [33-37]. In a further round of data analysis, we will also have the overtriaged cases assessed by panel B to include the low interrater reliability in the analysis. As with undertriaged cases, this is likely to reduce the overtriaged cases.

From the end of February, the COVID-19 pandemic required special hygiene measures for the tablet computers used, making patient recruitment more difficult as the first wave of the pandemic peaked in March 2020. The pandemic is also likely to have affected the case mix, which may have shifted slightly toward COVID-19–positive patients.

The urgency grading used in the 2D matrix for the triage levels (Table 1) was defined at the discretion of this study’s team, implicating a certain degree of subjectiveness. While the range of appropriate triage levels was defined based on this order, the experts did not always explicitly mark all of the intermediate triage levels as appropriate.

### Conclusions

The SMASS pathfinder symptom-checker proved to be a safe triage tool, avoiding undertriage in a real-life clinical setting of emergency consultations at a walk-in clinic and ED. Although for practical reasons the symptom-checker was not evaluated outside the hospital environment, our data do not suggest that its safety may have been compromised if used for self-triage by patients in a domestic setting.

## Abbreviations

ED: emergency department
ESI: Emergency Severity Index
SMASS: Swiss Medical Assessment System
WIC: walk-in clinic

## Data Availability

The raw data of our study is available from in4medicine, Inc [38].
